# 

**Published:** 1881-08

**Authors:** 


### Table of Contents.

ORIGINAL COMMUNICATIONS.

Hypodermic Medication for Beginners. By E. A. Cobleigh, M.D., Athens, Tenn........ 257
Preputial Deformities a Source of Serious Nervous Disturbances. By R. W. Westmoreland, M.D., Atlanta, Ga........ 264

REPORTS OF SOCIETIES.

Excision of the Tongue........ 268
Medical and Surgical Society of Baltimore........ 274
New York Surgical Society........ 280

SELECTIONS.

Iodoform as a Vermifuge........ 291
Four Cases of Tubercular Meningitis........ 294
Colchicum in Acute Rheumatism........ 307
Rare Cases of Syphilis........ 312

EDITORIAL.

A New Instrument for Extension and Counter-Extension in Injuries of the Lower Extremities........ 318
The Attempted Assassination........ 318
Publisher's Announcement........ 319
Meteorological Report........ 320

